# CRISPR-Cas-Induced Mutants Identify a Requirement for dSTIM in Larval Dopaminergic Cells of *Drosophila melanogaster*

**DOI:** 10.1534/g3.116.038539

**Published:** 2017-01-26

**Authors:** Trayambak Pathak, Deepti Trivedi, Gaiti Hasan

**Affiliations:** * National Centre for Biological Sciences, Tata Institute of Fundamental Research, Bangalore, Karnataka 560065, India; † Manipal University, Karnataka 576104, India

**Keywords:** null allele, Orai, SOCE, tyrosine hydroxylase, hypoderm

## Abstract

Molecular components of store-operated calcium entry have been identified in the recent past and consist of the endoplasmic reticulum (ER) membrane-resident calcium sensor STIM and the plasma membrane-localized calcium channel Orai. The physiological function of STIM and Orai is best defined in vertebrate immune cells. However, genetic studies with RNAi strains in *Drosophila* suggest a role in neuronal development and function. We generated a CRISPR-Cas-mediated deletion for the gene encoding STIM in *Drosophila* (*dSTIM*), which we demonstrate is larval lethal. To study STIM function in neurons, we merged the CRISPR-Cas9 method with the UAS-GAL4 system to generate either tissue- or cell type-specific inducible STIM knockouts (KOs). Our data identify an essential role for STIM in larval dopaminergic cells. The molecular basis for this cell-specific requirement needs further investigation.

The signaling properties of intracellular Ca^2+^ stores evolved in metazoans and plant cells where they regulate multiple biological processes including secretion, gene transcription, enzymatic activity, and motility (Prakriya and Lewis 2015). A range of external stimuli including hormones, neuromodulatory chemicals, and sensory signals activate their cognate membrane receptors resulting in cleavage of phosphatidyl inositol 1, 4 bisphosphate (PIP2) to generate inositol 1,4,5-trisphosphate (IP_3_), which in turn binds to an intracellular ligand-gated Ca^2+^ channel, the IP_3_ receptor (IP_3_R), present on ER Ca^2+^ store membranes (Patel *et al.* 1999; Clapham 2007). Release of ER Ca^2+^ through the IP_3_R generates transient cellular Ca^2+^ signals and simultaneously depletes intracellular Ca^2+^ stores. The drop in ER Ca^2+^ is sensed by ER membrane-localized STIM molecules through their Ca^2+^-binding EF hand motifs, leading to their clustering and rearrangement of the ER membrane with movement of the STIM clusters toward ER-PM junctions, where the physical interaction of STIM with the store-operated Ca^2+^ channel, Orai, results in extracellular Ca^2+^ entry generally referred to as store-operated Ca^2+^ entry or SOCE (Liou *et al.* 2007; Stiber *et al.* 2008). Unlike, the transient nature of Ca^2+^ release through the IP_3_R, SOCE can be sustained over minutes and hours and is likely to have significant and wide-ranging effects on organismal physiology and perhaps development. The kinetics of SOCE-derived cytosolic calcium signals are determined by the activity of a range of ionic exchangers, channels, and pumps, significant among which is the sarco-endoplasmic reticular Ca^2+^-ATPase (SERCA) pump (Sanyal *et al.* 2006; Venkiteswaran and Hasan 2009; Hasan and Venkiteswaran 2010; Brini *et al.* 2014).

For a better understanding of the role of SOCE during development and in organismal physiology, genetic studies of the key SOCE components STIM and Orai are required. Vertebrate studies have demonstrated that *STIM1*, *STIM2*, and *Orai1* KO mice are lethal (Baba *et al.* 2008; Gwack *et al.* 2008; Oh-Hora *et al.* 2008; Stiber *et al.* 2008; Beyersdorf *et al.* 2009), supporting an essential requirement for SOCE during vertebrate development. However, the underlying causes of lethality are not clearly understood. Unlike vertebrates, single genes encode the SOCE components *dSTIM* (CG9126) and *dOrai* (CG11430) in the fruit fly *Drosophila melanogaster* (Roos *et al.* 2005; Cai 2007). The study of *Drosophila dSTIM* and *dOrai* mutants can, thus, generate vital information about the nature of physiological and developmental processes regulated by SOCE. A hypomorphic allele of *dOrai*, *orai^3^*, has been studied previously for phagocytic function (Cuttell *et al.* 2008) and flight circuit development (Pathak *et al.* 2015), and has been described as partially lethal. However, mutants for *dSTIM* have not been characterized. Here, we have generated a complete KO as well as a tissue-specific Cas9-inducible UAS construct targeting the complete *dSTIM* open reading frame, using a modified CRISPR-Cas technique (Jinek *et al.* 2012; Cong *et al.* 2013; Mali *et al.* 2013). The inducible mutant strain allowed the investigation of multiple cell types and their individual contribution to the phenotype of the complete *dSTIM* KO. Surprisingly, lethality of *dSTIM* KO larvae was mirrored by inducing *dSTIM* mutations in cells expressing the enzyme tyrosine hydroxylase (TH), which include dopaminergic neurons and hypoderm cells.

## Materials and Methods

### Fly rearing and stocks

*Drosophila* strains were grown in standard corn flour agar media supplemented with yeast. All *Drosophila* strains were grown at 25°, unless specified in the text. Canton-S was used as the wild-type (*WT*) *Drosophila* strain. The ubiquitous GAL4 strain, *Actin5cGAL4* (BL4414); pan-neuronal drivers *Elav^C155^GAL4* (BL458) and *nSybGAL4* (BL51635); a muscle-specific GAL4, *Dmef2* (BL27390); a glutamatergic neuron GAL4, *OK371* (BL26160); and the *orai^3^* mutant strains (BL17538) were obtained from BDSC (Bloomington *Drosophila* Stock Center, Bloomington, IN). A peptidergic neuron GAL4, *c929*, was kindly gifted by P. H. Taghert, Washington University (Hewes *et al.* 2003). The *THGAL4* strain was a kind gift of S. Birman and has been described earlier (Friggi-Grelin *et al.* 2003). The *UASdOrai* (referred to as *dOrai*) and *UASdSTIM* (referred to as *dSTIM*) strains were generated by cloning of the publicly available full-length cDNA (RE30427) of *dOrai* and (BDGP LD45776) *dSTIM* (Venkiteswaran and Hasan 2009; Agrawal *et al.* 2010), followed by microinjection to obtain transgenic fly strains. The *THA*-GAL4 strain was kindly provided by M. Wu, Johns Hopkins University, Baltimore (Liu *et al.* 2012). The *UASCas9* strain (BL54593) was obtained from BDSC.

### Molecular cloning

Single guide (sgRNA) targets were designed at the 5′- and 3′-end of the STIM open reading frame (5′-sgRNA-dSTIM AATGCGAAAGAATACCATTTGG; 3′-sgRNA-dSTIM GGATGACTGAAGAACCTCTTGG). The sgRNAs were cloned in the *pU6-Bbs1-chiRNA* vector as previously described (Gratz *et al.* 2013). To make the dual-sgRNA constructs, the same two sgRNAs were cloned in *pBFv6.2* and *pBFv6.2B* vectors, respectively, and then a dual-sgRNA construct was generated as described by Kondo and Ueda (2013).

### Generation of dual-sgRNA transgenic flies

Dual-sgRNA was integrated into the *attP40* landing site on the second chromosome by phiC31 integrase using the *y1 v1 nos-phiC31*; *attP40* host (Bischof *et al.* 2007). Surviving G0 flies were intercrossed and the progeny was screened for the *v+* eye marker. A single transformant was mated to *y1*, *v1*; *Tft/CyO* flies. Offspring in which the transgene was balanced were collected to establish a stock.

### Generation of STIM KO

A total of 310 *w^1118^* embryos were injected with a mixture of plasmids encoding hsp70-Cas9 (Gratz *et al.* 2013) (500 ng/μl), *5′-sgRNA-STIM* (150 ng/μl), and *3′-sgRNA-STIM* (150 ng/μl). Seventy-two F0 adults that emerged were individually crossed with *FM7a* balancer flies. After 1 wk of egg laying, the parent F0 flies were individually squished in squishing buffer (10 mM Tris-HCl pH 8, 1 mM EDTA, 25 mM NaCl, and 200 g/ml Proteinase K) and were screened for deletion by PCR using the following primers: del-STIM-F, 5′-CTATGACTTTCGCGAGCAAC-3′ and del-STIM-R, 5′-CATCCGTTCCCTTCAGTTGT-3′.

Among the F0 flies, 17 individuals were identified as founders for the genomic deletion. A total of 198 F1 progeny obtained from these 17 founder lines were individually crossed to *FM7a* balancers. These were individually tested for deletion of the *dSTIM* locus by PCR using the primers mentioned above. Among these, two lines tested positive for deletion by PCR and were confirmed by sequencing of the PCR product. These heterozygous balanced flies were collected to establish a stock. Only one of the two lines was fertile and could be propagated further.

### Staging

Staging experiments were performed to obtain lethality profiles of the indicated genotypes as described previously (Joshi *et al.* 2004). Timed and synchronized egg laying was done for 6 hr at 25°. The larvae were collected at 60–66 hr after egg laying (AEL) in batches of 25 staged larvae. Each batch of 25 larvae was placed in a separate vial and minimally three vials containing agar-less media were tested for every genotype at each time point. Larvae were grown at 25°. Heteroallelic and heterozygous larvae were identified using dominant markers (*FM7iGFP*, *TM6Tb*, and *CyoGFP*). The larvae were screened at the indicated time points for number of survivors and stage of development, determined by the morphology of the anterior spiracles (Ashburner 1989). Experiments to determine the viability of experimental genotypes and their corresponding genetic controls were performed simultaneously in all cases.

### Data analysis and statistics

The size of third instar larvae was measured using Image J 1.50i (Wayne Rasband; Java 1.8.0_77). Size calibration was performed with a hemocytometer and sizes of third instar larvae were measured at the indicated times (AEL). Significant differences (*P < 0.05 and **P < 0.001) between data were tested by a Student’s *t*-test. Unless specified, all comparisons were to the WT. For larval staging experiments, significant differences were tested by Student’s *t*-test. The genotypes that were compared and the *P*-values obtained are shown in Table 3.

### Immunohistochemistry

Immunostaining of larval *Drosophila* brains was performed according to a published protocol for adult brains (Sadaf *et al.* 2015). The following primary antibodies were used at the indicated dilutions: anti-GFP antibody (1:10,000; A6455 Life Technologies) and Mouse anti-TH antibody (1:40; Immunostar). Secondary antibodies used were anti-rabbit Alexa Fluor 488 (#A1108, Life Technologies) and anti-mouse Alexa Fluor 568 (#A1104, Life Technologies) at a dilution of 1:400. Confocal images were obtained using an Olympus Confocal Microscope FV1000. Image visualization and analysis was performed using a FV10 ASW 4.2 viewer.

### RNA isolation and q-PCR

The method of RNA isolation and q-PCR was the same as described previously (Pathak *et al.* 2015), using the following primers: rp49 Forward, 5′-CGGATCGATATGCTAAGCTGT-3′; rp49 Reverse, 5′-GCGCTTGTTCGATCCGTA-3′; dSTIM Forward, 5′-GAAGCAATGGATGTGGTTCTG-3′; and dSTIM Reverse 5′-CCGAGTTCGATGAACTGAGAG-3′.

### Western blots

Larval or adult CNS of appropriate genotypes were dissected in cold PBS. Between 5 and 10 brains were homogenized in 50 µl of homogenizing buffer (25 mM HEPES pH 7.4, 150 mM NaCl, 5% Glycerol, 1 mM DTT, 1% Triton X-100, and 1 mM PMSF) and 10–15 µl of the homogenate was run on an 8% SDS-polyacrylamide gel. The protein was transferred to a nitrocellulose membrane by standard protocols and the membrane was incubated in the primary antibody overnight at 4°. Primary antibodies were used at the following dilutions: two mouse anti-dSTIM antibodies 8G1 and 3C1 (generated by Bioneeds, Bangalore, India) mixed at 1:1 and used at 1:20 dilution, and anti-β-tubulin monoclonal (E7, Developmental Studies Hybridoma Bank, University of Iowa, Iowa) at 1:5000. Secondary antibody conjugated with horseradish peroxidase was used at a dilution of 1:3000 (anti-mouse HRP; Cell Signaling 7076S). The protein was detected on the blot by a chemiluminescent detection solution from Thermo Scientific (No. 34075; Rockford, IL).

### Data availability

**Figure 1:**
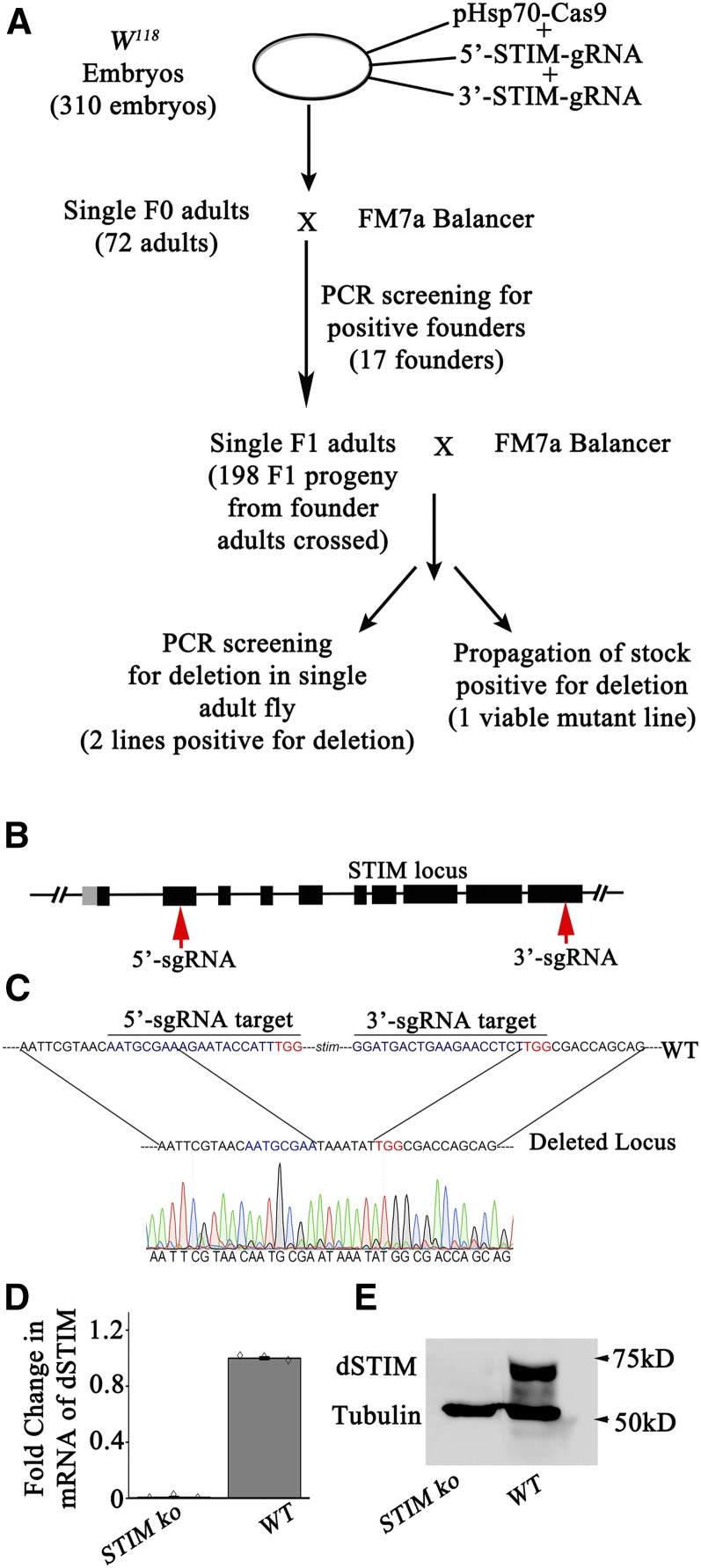
Knocking out *dSTIM* with the CRISPR-Cas9 system resulted in deletion of the *dSTIM* gene (A) Schematic representation for generation of STIM knockout (*STIMko*). Putative alleles were screened by PCR and balanced using first chromosome balancer FM7a. (B) Representation of *dSTIM* gene with exons (thick lines), introns (thin lines), and 5′−UTR (gray line). Target regions of guide RNAs are indicated with red arrows. (C) Sequencing of the *dSTIM* gene region confirming the deletion. (D) q-PCR of RNA isolated from *STIMko* second instar larvae (*n* = 3). The error bars represent SEMs. (E) Western blot of protein lysates from second instar larva of *STIMko* organisms. CRISPR, clustered regularly interspaced short palindromic repeats; PCR, polymerase chain reaction; q-PCR, quantitative PCR; UTR, untranslated region; WT, wild-type.

**Figure 2:**
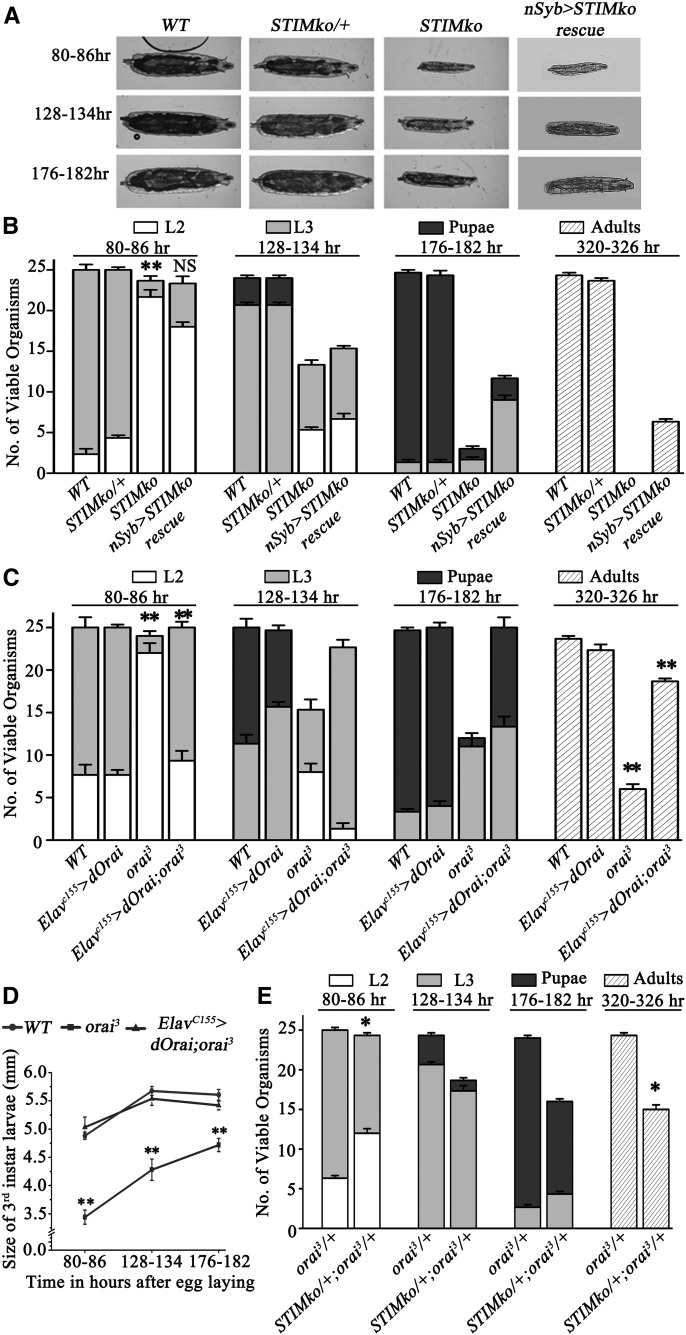
*dSTIM* knockout organisms are third instar larval lethal. (A) Larval images represent size and stage at indicated times in hours AEL. (B) The bar graph represents average number of viable organisms at the indicated time in hours AEL (± SEM). Each bar represents number of viable organisms (out of 25 organisms) and their stage of life cycle. L2 stands for second and L3 for third instar larval stage, respectively. *STIM* knockout (*STIMko*) organisms die as late second or early third instar larvae and exhibit slow growth, which was partially rescued by pan-neuronal overexpression of *dSTIM* (*nSyb > STIMko* rescue), **P < 0.001. (C) *orai^3^* homozygotes lag behind in development and start dying as third instar larvae. Pan-neuronal over expression of *dOrai* (*Elav^C155^ > dOrai;orai^3^*) rescued both larval lethality and slow growth of *orai^3^* homozygotes. (D) Line graph represents third instar larval size at particular time points. Larvae of *orai^3^* homozygotes were smaller compared to controls. The reduced size of *orai^3^* homozygotes was rescued by pan-neuronal *dOrai* expression (*n* = 3*10, Student’s *t*-test **P < 0.001). (E) Heteroallelic combination of *STIMko* and *orai^3^* (*STIMko/+;orai^3^/+*) showed partial lethality, *P < 0.05. Numbers of second instar larvae at 80–86 hr and adults at 320–326 hr were compared by the Student’s *t*-test. The genotypes compared, with their *P*-values, are given in Table 3. AEL, after egg laying; NS, not significant; WT, wild-type.

**Figure 3:**
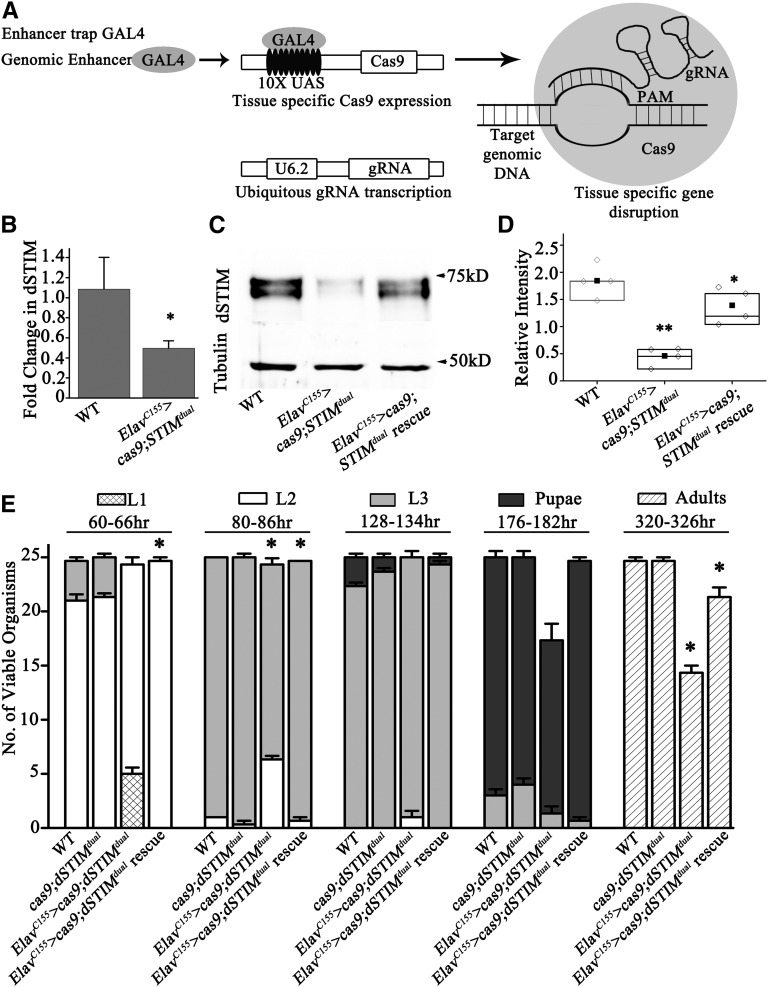
Pan-neuronal knock out of *dSTIM* leads to lethality of third instar larvae. (A) Schematic representation of the method for knocking out *dSTIM* from specific cells and/or tissues. *Cas9* is under the *UAS* promoter expression, which is controlled by GAL4. Expression of the gRNA pair is driven across all tissues by the U6.2 promoter. (B) q-PCR with RNA isolated from the CNS of third instar larvae of the indicated genotypes. *Elav^C155^ > cas9;STIM^dual^* (*n* = 3, *P < 0.05) show reduced *dSTIM* transcript levels as compared to WT controls. (C) A representative western blot from the CNS of *Elav^C155^ > cas9;STIM^dual^* organisms showing reduced levels of dSTIM protein. The CNS lysate from *Elav^C155^ > cas9;STIM^dual^*;*dSTIM* (rescue) organisms exhibits higher dSTIM protein expression as compared with *Elav^C155^ > cas9;STIM^dual^* organisms. (D) Quantification of the relative intensity of dSTIM bands as compared with tubulin (*n* = 3, Student’s *t*-test **P < 0.001, *P < 0.05). (E) Staging of animals with pan-neuronal knockout of *dSTIM* (*Elav^C155^ > STIM^dual^*) and their rescue by overexpressing *dSTIM* (*Elav^C155^ > cas9*; *STIM^dual^*; *dSTIM*). The *Cas9* transgene is present in all organisms except WT. Error bars represent SEMs. Numbers of second instar larvae at 80–86 hr and adults at 320–326 hr were compared by the Student’s *t*-test. The genotypes compared, with their *P*-values, are given in Table 3. CNS, central nervous system; gRNA, guide RNA; q-PCR, quantitative PCR; WT, wild-type.

**Figure 4:**
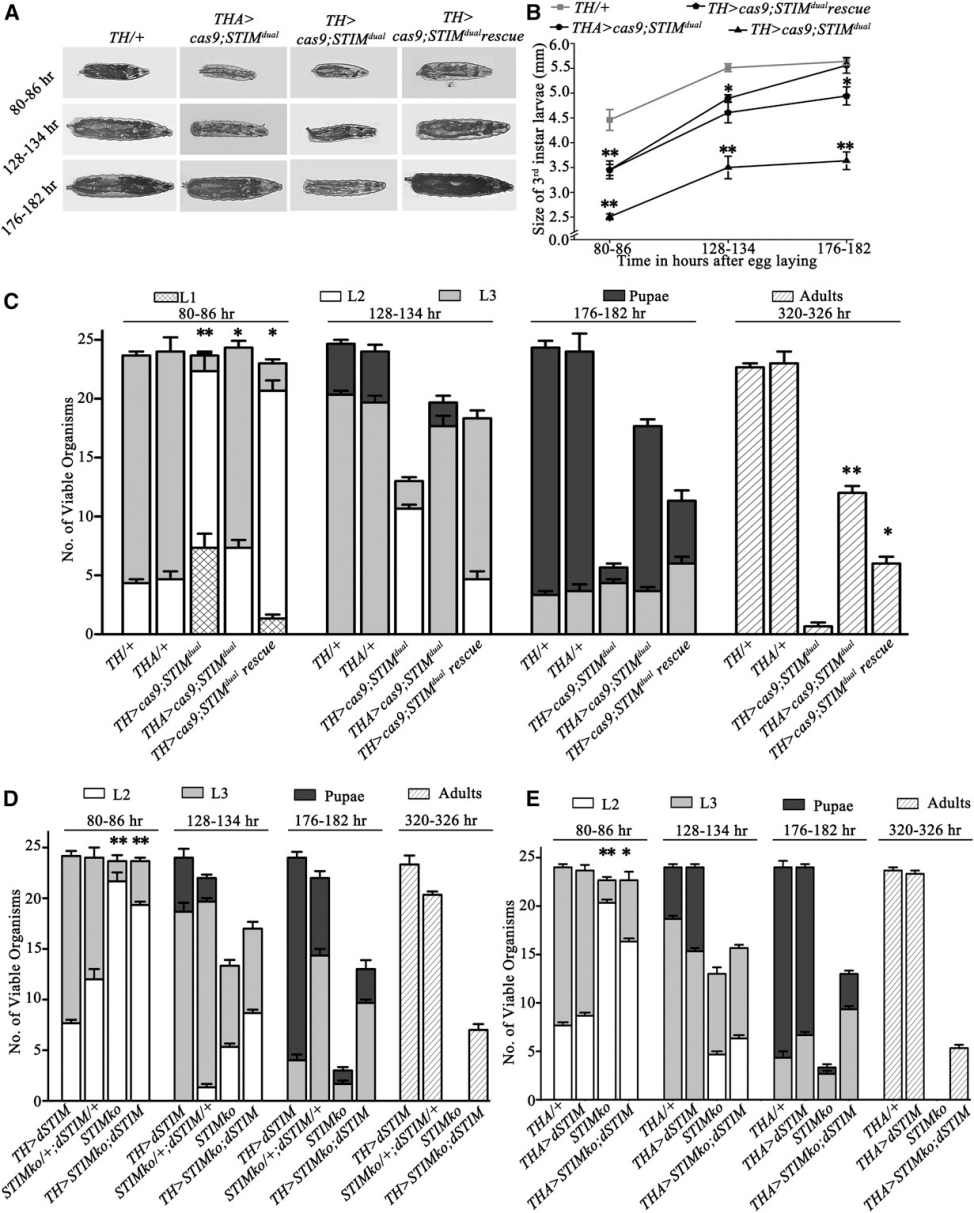
Knock out of the d*STIM* gene from dopaminergic cells resulted in larval lethality. (A) Larval images at the indicated time in hours AEL. *TH > cas9;STIM^dual^* organisms were smaller as compared to control *THGAL4* heterozygotes (*TH/+*). (B) The line graph represents size of third instar larvae at indicated time points. *TH > cas9;STIM^dual^* were smaller as compared to *THGAL4* heterozygotes (*TH/+*). Overexpression of *dSTIM* (*TH > cas9;STIM^dual^* rescue) partially rescued the larval size compared to *TH > cas9;STIM^dual^* organisms. *dSTIM* knockout from the hypoderm (*THA > cas9;STIM^dual^*) also resulted in reduced larval size at 80–86 hr and 128–134 hr AEL compared to *TH/+* organisms (*N* = 10, Student’s *t*-test *P < 0.05, **P < 0.001). (C) *TH > cas9;STIM^dual^* and *THA > cas9;STIM^dual^* organisms were lethal at larval stage. They were partially rescued by *dSTIM* overexpression in dopaminergic cells. (D) Overexpression of *dSTIM* (*UASdSTIM*) in dopaminergic neurons (*TH > STIMko;dSTIM*) partially rescued the larval lethality of *STIMko* organisms. The *STIMko* data are as in Figure 2B and are included here for ease of comparison. Both experiments were performed simultaneously. (E) Overexpression of *dSTIM* in TH-producing cells of the cuticle (*THA > STIMko;dSTIM*) partially rescued the larval lethality of *STIMko* organisms. The number of adults of *TH > STIMko;dSTIM* and *THA > STIMko;dSTIM* was not compared to *STIMko* because *STIMko* organisms were lethal. Numbers of second instar larvae at 80–86 hr and adults at 320–326 hr were compared by the Student’s *t*-test. The genotypes compared, with their *P*-values, are given in Table 3. AEL, after egg laying; TH, tyrosine hydrolase.

There are six supplemental files associated with this manuscript. Data in these supplemental files supports the results of the main figures and text. Supplemental material File S1 contains the detailed figure legends for all the Supplemental figures. Figure S1 contains supporting data for Figure 1 with the results of PCR screening for identifying putative STIMko strains. Figure S2 has genetic controls for the data with *orai^3^* mutants and *STIMko* presented in Figure 2. Figure S3 contains supporting data and genetic controls for Figure 3. Genetic controls and data supporting a role for dOrai in larval dopaminergic neurons is shown in Figure S4, whereas further data supporting a role of dSTIM in larval dopaminergic neurons is presented in Figure S5. Both Figure S4 and Figure S5 support the results presented in Figure 4.

## Results

### Generation of a KO for dSTIM using the CRISPR-Cas9 system

A null allele for *dSTIM* was generated with the CRISPR-Cas9 methodology (Jinek *et al.* 2012; Cong *et al.* 2013; Gratz *et al.* 2013; Mali *et al.* 2013; Ren *et al.* 2013). For this purpose, two sgRNAs that were individually designed to target double-stranded breaks at the 5′- and 3′- ends of the *dSTIM* gene were introduced in *Drosophila* embryos with a plasmid encoding Cas9 (see *Materials and Methods* for details). Animals with a deficiency of the complete coding region of the *dSTIM* gene (Figure 1B) were identified by a PCR strategy after obtaining DNA from heterozygotes at the F1 generation (Figure 1A and Figure S1A). Approximately 198 FM7a balanced heterozygous flies were screened by PCR, from which we obtained two flies with a putative deletion for *dSTIM* (Figure 1A and Figure S1B). The *dSTIM* deletion was confirmed by sequencing of genomic DNA obtained from larvae of *dSTIM* KO homozygotes (*STIMko*, Figure 1C). To further confirm the *dSTIM* deletion, q-PCRs and westerns were performed from RNA and protein isolated from the second instar larvae of *STIMko* organisms. *dSTIM* transcripts were undetectable in *STIMko* organisms as compared to controls (Figure 1D). Concurrent with this, dSTIM protein was undetectable in *STIMko* organisms (Figure 1E).

### Whole-body KO of dSTIM and orai^3^ homozygotes are larval lethal

To begin understanding the functional significance of reduced SOCE during *Drosophila* development, the viability of homozygous *STIMko* organisms was determined. Complete *STIMko* organisms were consistently smaller (Figure 2A) and did not survive beyond late second or early third instar larval stages (Figure 2B). *STIMko* homozygotes began to exhibit a delay in development from 80 to 86 hr AEL. Among the batches of 25 larvae counted from *STIMko* organisms, 21.6 ± 0.8 sec instar and 2 ± 0.5 third instar were observed at 80–86 hr, indicating a significant delay in larval development when compared to *STIMko* heterozygotes (*STIMko*/+) of which just 4.3 ± 0.3 remained as second instar, whereas 20.6 ± 0.3 had progressed to the third instar larval stage. From 128 to 134 hr onwards, *STIMko* organisms exhibited lethality. Viable *STIMko* organisms were present as either second instar (5.3 ± 0.3) or third instar larvae (8 ± 0.5; Figure 2B). In contrast, control *STIMko*/+ organisms, had progressed to either late third instar larvae (20.6 ± 0.3) or pupae (2.6 ± 0.3; Figure 2B). At 176–182 hr, the majority of *STIMko* organisms were dead whereas controls were mostly pupae (23.3 ± 0.5) and very few were third instar larvae (1.3 ± 0.3) (Figure 2B). Because CRISPR-Cas9 can introduce mutations at nontarget sites, the ability of a *dSTIM* transgene (*UASdSTIM*; Agrawal *et al.* 2010; Port *et al.* 2014) to rescue lethality in *STIMko* organisms was tested by expression with a ubiquitously expressed GAL4 (*Actin5cGAL4*). More than 80% *STIMko* animals were rescued (20.3 ± 0.3) and emerged as adults by ubiquitous expression of *dSTIM*, indicating that lethality in the CRISPR-Cas9-generated KO strain was primarily due to loss of the *dSTIM* gene (Figure S2F).

The best understood cellular role for STIM is in activation of the store-operated Ca^2+^ channel, Orai, after depletion of intracellular Ca^2+^ stores (Feske *et al.* 2006; Prakriya *et al.* 2006; Vig *et al.* 2006; Zhang *et al.* 2006). However, instances where STIM can interact with and activate Ca^2+^ channels other than Orai are also known (Soboloff *et al.* 2006; Brandman *et al.* 2007; Park *et al.* 2010; Wang *et al.* 2010; Nguyen *et al.* 2013). To understand if lethality in *STIMko* organisms is a consequence of reduced SOCE through dOrai, viability of *orai^3^* larvae was measured. *orai^3^* is a previously described hypomorphic allele of *dOrai* (Cuttell *et al.* 2008; Pathak *et al.* 2015). Concurrent with lethality of *STIMko* larvae, *orai^3^* homozygotes were 80% lethal as third instar larvae (Figure 2C). Moreover, *orai^3^* homozygous larvae were smaller in size as compared to control (*WT*) organisms (Figure 2D and Figure S2A). However, unlike *STIMko* organisms, a greater number of *orai^3^* larvae could pupate (∼25%). *orai^3^* pupae were viable and eclosed as adults (Figure 2C). Both *orai^3^* homozygous larvae and adults appeared smaller in size as compared to CS controls (Figure 2D and Figure S2, A and C). If lethality of both *STIMko* and *orai^3^* larvae is indeed a consequence of reduced SOCE through Orai, we predicted that single copies of *STIMko* and *orai^3^* in the same organism should exhibit lethality. Indeed, *STIMko/+*; *orai^3^/+* females are partially inviable (15.0 ± 0.6; Figure 2E), as compared to individual heterozygotes where no significant lethality was observed (Figure 2, B and E).

Next, to test the tissue-specific requirement for SOCE in *Drosophila* larvae, rescue of *STIMko* and *orai^3^* organisms was tested by overexpression of cDNAs encoding either *dSTIM* or *dOrai*. Pan-neuronal overexpression of *dSTIM* with *nSybGAL4* did not rescue larval size (Figure 2A) and slower development (Figure 2B), but it did rescue lethality of *STIMko* organisms to an extent (6.3 ± 0.3 adults; Figure 2B). Residual lethality of *nSyb* > *STIMko*;*UASSTIM* organisms was at late second and third instar larval stages (Figure 2B). All *nSyb* > *STIMko*;*UASSTIM* pupae eclosed as adults, supporting a requirement for SOCE in the development of the nervous system in pupae (Pathak *et al.* 2015).

A requirement for SOCE in postembryonic development of the nervous system was reiterated upon pan-neuronal expression of *dOrai* with *Elav^C155^GAL4* in *orai^3^* homozygotes (Figure 2C). Whereas just 6 ± 0.5 homozygous *orai^3^* organisms survive to adulthood, ∼80% of rescued larvae eclosed as adults (18.6 ± 0.3) after normal progression through larval stages. Rescued *orai^3^* homozygous larvae appeared normal in size (5.4 ± 0.07 mm at 176–182 hr) and as adults (2.68 ± 0.03 mm) (Figure 2D and Figure S2, A and C). Control animals including *orai^3^* heterozygotes and animals with pan-neuronal overexpression of *dOrai* (*Elav^C155^ > dOrai*) were viable and developed normally (Figure S2B). These results confirmed that lethality, slow development, and smaller body size of hypomorphic *orai^3^* homozygotes arises primarily from reduced SOCE through dOrai in neurons. The function of dSTIM in neurons and other tissues appeared more complex and was investigated further.

The difference in the extent of rescue by the transgenes encoding WT *dSTIM* and *dOrai* (compare Figure 2, B and C) could arise due to differences in GAL4-driven expression from the *Elav^C155^* and *nSyb* promoters (Figure S2E). In addition, the differential rescue may be attributed to the fact that residual expression of dOrai is seen in *orai^3^* homozygous larvae (a hypomorph; Figure S2D), whereas dSTIM protein is not detectable in *dSTIMko* larvae (Figure 1E).

### Characterization of tissue-specific STIMko

To understand *dSTIM* function in specific tissues, a modified CRISPR-Cas9 system was developed. For this purpose, a transgenic strain was generated where both 5′- and 3′-guide RNAs for *dSTIM* (*STIM^dual^*) express under control of the ubiquitous U6.2 promoter (Kondo and Ueda 2013; Xue *et al.* 2014) (Figure 3A). Next, we placed the *STIM^dual^* strain with a *UAS-Cas9* transgene (Port *et al.* 2014). The resultant flies were mated with specific GAL4 strains to generate cell and tissue-specific KOs of *dSTIM* (Figure 3A). To test this system, *Elav^C155^GAL4*-driven pan-neuronal expression of *Cas9* with *STIM^dual^* was attempted. The level of *dSTIM* transcripts were reduced significantly in the CNS of *Elav^C155^ > cas9;STIM^dual^* organisms (Figure 3B), and dSTIM protein was also reduced to slightly less than half of WY levels (Figure 3, C and D). These results confirmed that the cell-specific KO of *dSTIM* with CRISPR-Cas9 reduced *dSTIM* transcript and protein levels significantly. Residual *dSTIM* transcripts and protein could be due to a number of reasons. First, some may derive from nonneuronal and neuronal cells in the brain that express dSTIM but do not express *Elav^C155^GAL4*. Second, the method of tissue-specific KO employed here may not drive complete excision of both *dSTIM* alleles in all *GAL4*-expressing cells, as evident from recently published studies (see *Discussion* and Port *et al.* 2014; Moreno-Mateos *et al.* 2015).

To further investigate whether the reduced viability of the *dSTIM* KO strain is due to its function in neurons, the development and viability of *Elav^C155^ > cas9;STIM^dual^* organisms was investigated. Pan-neuronal KO of *dSTIM* led to partial lethality of third instar larvae as compared to control *cas9;STIM^dual^* or *WT* organisms. Similar to *STIMko* organisms, *Elav^C155^ > cas9;STIM^dual^* organisms also develop slowly. At 60–66 hr after AEL, 5.5 ± 0.5 organisms were first instar larvae and 19.6 ± 0.6 were second instar, as compared to controls where all organisms were second instar or third instar. At 80–86 hr, the number of second and third instar larvae in *Elav^C155^ > cas9;STIM^dual^* organisms were 6.3 ± 0.3 and 18.5 ± 0.3, respectively, indicating that although second and third instar larvae were not lethal yet, their development was slower as compared to control organisms (Figure 3E). Similarly, at 128–134 hr, *Elav^C155^ > cas9;STIM^dual^* organisms persisted as second instar (1 ± 0.5) and third instar (24 ± 0.5) larvae, whereas control *cas9;STIM^dual^* organisms were either third instar (23 ± 0.3) or pupae (1.3 ± 0.3; Figure 3E). At 176–184 hr, fewer *Elav^C155^ > cas9;STIM^dual^* organisms were observed and these were in third instar (1.3 ± 0.6) and pupal (16 ± 0.5) stages. Control *cas9;STIM^dual^* organisms were also present as third instar larvae (4 ± 0.5) and pupae (21 ± 0.5) at this time (Figure 3E). These data demonstrate that *Elav^C155^ > cas9;STIM^dual^* organisms were partially lethal as third instar larvae.

*Elav^C155^ > cas9;STIM^dual^* organisms that pupated also eclosed as adults (14.3 ± 0.6; Figure 3E). However, all eclosed organisms were females, indicating that *Elav^C155^ > cas9;STIM^dual^* organisms were male lethal. The difference in lethality between males and females needs further investigation. Interestingly, overexpression of *dSTIM* in *Elav^C155^ > cas9;STIM^dual^* organisms rescued their developmental delay, but lethality was rescued only partially (21.3 ± 0.8 eclosed adults; Figure 3E). The difference between rescue of developmental delay and lethality may be due to incomplete rescue of dSTIM expression in the CNS of *Elav^C155^ > cas9;STIM^dual^*; *dSTIM* organisms (Figure 3, C and D). It is possible that the threshold for rescue of developmental delay by dSTIM is lower, whereas it is higher for rescue of lethality.

Similarly, KO of *dSTIM* with another pan-neuronal driver (*nSybGAL4*) resulted in larval lethality. *nSyb > cas9;STIM^dual^* organisms were also lethal as third instar larvae. Both males and females were present among the adults that eclosed (15.3 ± 1.2; Figure S3). Pan-neuronal overexpression of *dSTIM* or *cas9* alone did not result in lethality (Figure S3). In contrast to *STIMko* organisms, the larval size of *Elav^C155^ > cas9;STIM^dual^* organisms was similar to controls. Thus, *dSTIM* function is required in neurons of late second and third instar larvae for viability of *Drosophila*. The absence of growth deficits in *Elav^C155^ > cas9;STIM^dual^* organisms could be due to partial KO (Figure 3, B–D). Alternatively, the growth deficits in *STIMko* might be due to a requirement for dSTIM in nonneuronal tissues where *Elav^C155^ GAL4* does not express.

### SOCE is required in dopaminergic cells for viability of Drosophila

Next, we investigated the classes of neurons that require dSTIM/dOrai function for larval viability. Overexpression of *dOrai* in either dopaminergic (*THGAL4*) or glutamatergic (*OK371GAL4*) neurons of *orai^3^* homozygotes resulted in partial rescue of lethality and delay in larval development (Figure S4, A and B). Rescue of lethality from dopaminergic neurons was better (13 ± 0.6 adults) as compared with glutamatergic neurons (9 ± 0.6 adults). Lethality of *orai^3^* homozygous larvae was not rescued by overexpression of *dOrai* in either peptidergic neurons (*c929GAL4*) or muscles (*Dmef2GAL4*) (Figure S4, C and D). These data suggest that SOCE through the STIM/Orai pathway is required in dopaminergic cells for larval viability. This idea was tested further by investigating lethality and developmental profiles of *TH > cas9;STIM^dual^* organisms with KO of *dSTIM* in dopaminergic cells. *TH > cas9;STIM^dual^* organisms were developmentally delayed as evident from the presence of first instar larvae (7.3 ± 1.2) at 80–86 hr AEL and the persistence of second instar larvae (10.6 ± 0.3) at 128–134 hr AEL (Figure 4C). In addition, *TH > cas9;STIM^dual^* organisms are almost completely inviable. The average number of adults that eclosed from *TH > cas9;STIM^dual^* organisms was 0.6 ± 0.3 (Figure 4C). Overexpression of WT *dSTIM* in a background of *TH > cas9;STIM^dual^* organisms partially rescued lethality (6 ± 0.5 adults) and developmental delay (Figure 4, A–C). To understand why greater lethality was observed in *TH > cas9;STIM^dual^* organisms as compared with *Elav^C155^ > cas9;STIM^dual^* organisms, we analyzed the extent of overlap between TH- and Elav-expressing neurons in post 80–86-hr-old larval brains. Indeed, at this stage of brain development the overlap of TH-positive cells and *Elav^C155^ > GFP* -positive cells was incomplete, both in the brain lobes (Figure S5D) and the ventral ganglion (data not shown). For example, *Elav^C155^ > GFP* expression was present in the DM cluster of TH-positive cells, but the DL1 and DL2 clusters (Friggi-Grelin *et al.* 2003) were not marked by *Elav^C155^* *>* GFP (Figure S5D). These data do not rule out the possibility of *Elav^C155^*-driven expression of *UAScas9* in all TH-expressing cells at earlier stages of larval development, possibly resulting in early targeting of the *dSTIM* locus by the *STIM^dual^* transgene. However, they do establish the lack of a complete overlap between expression of *Elav^C155^GAL4* and *THGAL4* through all developmental stages of the larval CNS. Thus, even though *Elav^C155^GAL4* expresses in many more cells and expression of *THGAL4* is restricted to fewer cells (compare Figure S2E and Figure S5D), there is apparently a greater requirement for dSTIM in cells expressing TH. However, this requirement does not result in a loss of TH cells. When TH cells were counted in larval brains from *TH > mGFP* and *TH > mGFP*; *cas9;STIM^dual^* organisms at 80–86 hr AEL and 176–182 hr AEL, the numbers obtained from both genotypes matched published data (Friggi-Grelin *et al.* 2003; Figure S5C).

It is known that, in addition to expression in neurons, TH (encoded by *TH*) is expressed in the hypodermal cells and is required for melanization (Marsh and Wright 1980; Wright 1987; Birman *et al.* 1994). To understand if the loss of viability in *TH > cas9;STIM^dual^* organisms arises as a consequence of loss of dSTIM function in the hypoderm, we tested viability of *THA > cas9;STIM^dual^* organisms. The *THA-GAL4* strain does not express in TH neurons (Liu *et al.* 2012 and Figure S5A). *THA > cas9;STIM^dual^* organisms were partially lethal at late second and early third instar larval stages but did not appear developmentally delayed (Figure 4C). However, *THA > cas9;STIM^dual^* organisms were smaller in size compared to *THA/+* control organisms (Figure 4, A and B). The extent of larval lethality was significantly less as compared with *TH > cas9;STIM^dual^* organisms, and there was no pupal lethality (Figure 4C). These data suggest a significant requirement for dSTIM function and SOCE in larval dopaminergic cells in the brain and in the hypoderm. A possible reason for lethality of *TH > cas9;STIM^dual^* larvae could be that dSTIM in hypodermal TH cells is required for the formation of mouth hooks, in turn essential for feeding. However, mouth hooks of *TH > cas9;STIM^dual^* and *THA > cas9;STIM^dual^* organisms appeared normal (Figure S5B). Next, we tested the extent of rescue of *STIMko* organisms by overexpression of *dSTIM* in dopaminergic cells. Indeed, a partial rescue was observed. A few *TH > STIMko;dSTIM* (7 ± 0.5) and *THA > STIMko;dSTIM* (5.3 ± 0.3) organisms eclosed as adults as compared to *STIMko* organisms that were completely lethal (Figure 4, D and E). However, *STIMko* organisms with overexpression of *dSTIM* in TH-expressing cells (either with *TH* or *THA-GAL4*) continued to exhibit significant larval lethality and developmental delays (Figure 4, D and E). Thus, dSTIM function is required in dopaminergic cells but expression in dopaminergic cells alone is insufficient for complete rescue of viability. These data confirm a role for SOCE in dopaminergic neurons for the development and viability of larvae, and support a novel role for dSTIM in TH-expressing cells of the hypoderm.

## Discussion

The two major components of SOCE, STIM and Orai, have been implicated in both vertebrate and invertebrate development. In this study, we generated a complete KO for the *dSTIM* gene, as well as a modified inducible version, so as to understand the role of dSTIM in the development and viability of *Drosophila*. To generate *STIMko* animals, we adopted the CRISPR-Cas9 technique and screened for putative heterozygous *STIMko* founders by PCR. A comparison of the phenotypes of *STIMko* organisms with an existing *orai* hypomorphic allele established that SOCE is required during second and early third instar for viability. Results from a combination of rescue experiments, plus an inducible strain designed for generating *dSTIM* KOs, demonstrate that a major focus of SOCE requirement are dopaminergic neurons in the CNS (Table 1, Table 2 and Table 3). This is significant because dopaminergic neurons are known to regulate multiple aspects of neuronal physiology and behavior in mammals and *Drosophila* (Schultz 2007; Yamamoto and Seto 2014). In addition, dSTIM function may be required in nonneuronal cells for growth and viability. It remains to be established if all phenotypes associated with the KO of *dSTIM* arise as a consequence of loss of SOCE through Orai or from the ability of STIM to regulate other channels including the voltage-gated Ca^2+^ channels (Harraz and Altier 2014). A role for SOCE in the larval CNS agrees with previous findings of IP_3_R mutants (Joshi *et al.* 2004), and more recent studies demonstrating that the IP_3_R regulates SOCE in *Drosophila* neurons (Venkiteswaran and Hasan 2009; Chakraborty *et al.* 2016; Deb *et al.* 2016). The precise target(s) of SOCE in the larval nervous system and in nonneuronal cells needs further investigation.

**Table 1:** Comparison of lethality, developmental delay, and body size on knocking out *dSTIM* or reducing *dOrai* function

Genotype	Percentage Lethality	Developmental Delay	Small Body Size
*STIMko*	100	++	++
*orai^3^*	80	++	++
*Elav^C155^ > cas9;STIM^dual^*	45	+	—
*nSyb > cas9;STIM^dual^*	40	+	—
*TH > cas9;STIM^dual^*	98	++	++
*THA > cas9;STIM^dual^*	50	+	+

+ , strength of phenotype; —, no phenotype; TH, tyrosine hydrolase.

**Table 2:** Comparison of rescue of lethality, developmental delay, and body size on overexpressing *dSTIM* or *dOrai* in background of *dSTIM* knockout or *orai^3^*, respectively

Genotype	Percentage Rescue of Lethality	Rescue of Developmental Delay	Rescue of Body Size
*nSyb > STIMko;dSTIM*	20	—	—
*TH > STIMko;dSTIM*	30	+	—
*THA > STIMko;dSTIM*	20	+	—
*TH > cas9;STIM^dual^;dSTIM*	25	+	++
*Elav^C155^ > orai^3^;dOrai*	80	++	++
*nSyb > orai^3^;dOrai*	45	++	++
*TH > orai^3^;dOrai*	45	++	++
*OK371 > orai^3^;dOrai*	10	+	+
*c929 > orai^3^;dOrai*	—	—	—
*dMef2 > orai^3^;dOrai*	—	—	—

—, no rescue; +, strength of rescue; TH, tyrosine hydrolase.

**Table 3:** Comparison of number of second instar larvae at 80–86 hr and adults at 320–326 hr

Figure	Genotype	Compared to	*P* value
Figure 2B	*STIMko*	*STIMko/*+	< 0.001
	*nSyb > STIMko rescue*	*STIMko*	NS
Figure 2C	*orai^3^*	*WT*	< 0.001
	*Elav^C155^ > dOrai*; *orai^3^*	*orai^3^*	< 0.001
Figure 2E	*STIMko/*+*orai^3^/*+	*orai^3^/*+	< 0.05
Figure 3E	*Elav^C155^ > cas9;STIM^dual^*	*cas9;STIM^dual^*	< 0.05
	*Elav^C155^ > cas9;STIM^dual^* rescue	*Elav^C155^ > cas9;STIM^dual^*	< 0.05
Figure 4C	*TH > cas9;STIM^dual^*	*TH/*+	< 0.001
	*THA > cas9;STIM^dual^*	*THA/*+	< 0.05
	*TH > cas9;STIM^dual^ rescue*	*TH > cas9;STIM^dual^*	< 0.05
Figure 4D	*STIMko*	*STIMko/*+*;dSTIM/*+	< 0.001
	*TH > STIMko;dSTIM*	*STIMko*	< 0.001
Figure 4E	*STIMko*	*THA/*+	< 0.001
	*THA > STIMko;dSTIM*	*STIMko*	< 0.001
Figure S4A	*orai^3^*	*TH/*+	< 0.001
	*TH > dOrai;orai^3^*	*orai^3^*	< 0.05
Figure S4B	*orai^3^*	*OK371/*+	< 0.05 and < 0.001
	*OK371 > dOrai;orai^3^*	*orai^3^*	< 0.05
Figure S4C	*orai^3^*	*c929/*+	< 0.05 and < 0.001
	*c929 > dOrai;orai^3^*	*orai^3^*	< 0.05
Figure S4D	*orai^3^*	*dMef2/*+	< 0.05 and < 0.001
	*Dmef2 > dOrai;orai^3^*	*orai^3^*	< 0.05

NS, not significant; WT, wild-type; TH, tyrosine hydrolase.

Interestingly, complete lethality, developmental delays, and growth deficits of *STIMko* larvae and pupae was replicated closely by specific targeting of the *dSTIM* locus in dopaminergic cells. Indeed, pan-neuronal targeting of the *dSTIM* locus resulted in only 40–45% lethality, suggesting that dopaminergic cells are especially susceptible to loss of SOCE. An alternate explanation for differences between the extent of lethality observed in organisms when STIM*^dual^*; *cas9* is driven by either *Elav^C155^GAL4* or *THGAL4* could be a low level of differential leaky *GAL4* expression in nonneuronal tissues, and therefore “nonspecific” expression of the *UAScas9* construct in the two strains. At present, we are unable to resolve this issue, but based on the stronger phenotype of *TH > STIM^dual^* *vs.* *Elav^C155^ > STIM^dual^* (compare Figure 3E with Figure 4C) this seems unlikely, because visible nonspecific expression of *THGAL4* (as viewed by driving *UASmGFP* in early third instar larvae; Figure S5A) is considerably more restricted in the whole animal as compared to expression of *Elav^C155^GAL4* (data not shown). It should be possible to address this more rigorously in future by generating fluorescently-marked *dSTIM* alleles, allowing for visualization of loss of one or both alleles in any tissue of interest.

The difference in lethality observed between *Elav^C155^GAL4*- and *THGAL4*-driven *STIM^dual^* could in part also arise due to differential efficiency of tissue-specific mutagenesis in the two *GAL4* strains. It should be possible to address this issue in future by using a strain that creates *dSTIM* KOs at a higher efficiency. The *STIM^dual^* strain used in this study targets two sites at the ends of the *dSTIM* open reading frame with the idea that they should create a complete KO (see *Materials and Methods*). The detectable presence of dSTIM transcripts and protein in larval brain lysates of *Elav^C155^GAL4 > STIM^dual^;cas9* suggests that a complete KO of both alleles may not occur in all neuronal cells marked by *Elav^C155^GAL4*, though some of the residual transcripts and protein could arise from nonneuronal cells, such as glia that do not express *Elav^C155^GAL4*. Recent studies suggest that increasing the target sites to three or more within a locus is a more dependable strategy for obtaining tissue-specific KOs (Sunagawa *et al.* 2016).

Nevertheless, a critical requirement for SOCE in dopaminergic cells is supported by earlier results with cell- and tissue-specific knockdown of SOCE components (Pathak *et al.* 2015). Whereas the KO data (Table 1) strongly implicate dopaminergic cells as the focus of SOCE requirement in larvae, the rescue experiments (Table 2) also support a pan-neuronal requirement for dSTIM and dOrai. Pan-neuronal (*Elav^C155^GAL4*) overexpression of *dOrai* rescued lethality of *orai^3^* homozygotes to a greater extent than overexpression from dopaminergic cells alone (Figure 2B, Figure S4A, and Table 2), though developmental delays and size were rescued to similar extents. However, in *STIMko* organisms, both pan-neuronal and dopaminergic overexpression led to a partial and comparable level of rescue of lethality (Figure 2B, Figure 4D, and Table 2). We attribute these differences to the hypomorphic nature of the *orai^3^* allele, as compared to d*STIMko* which is a null allele. Differential expression patterns of *Elav^C155^GAL4* and *nSybGAL4* (Figure S2E) may also contribute to the difference in rescue of *orai^3^* and *STIMko* organisms. Rescue of *STIMko* was attempted with *nSybGAL4* because the *Elav^C155^GAL4* transgene and *STIMko* are both on the X chromosome. Despite the near complete lethality of *TH > cas9*; *STIM^dual^* larvae, *TH*-driven rescue of *STIMko* organisms remained at 30% (Table 1 and Table 2). *dSTIM* expression in dopaminergic cells is, thus, not sufficient for complete viability of *STIMko* animals, and indicates a requirement in other neuronal subdomains and tissues. Previously, it was demonstrated that SOCE is required for the regulation of *TH* gene transcription in pupae (Pathak *et al.* 2015). The larval requirement for SOCE in dopaminergic cells may be similar, though effects of SOCE on cellular processes other than gene regulation remain a possibility.

## Supplementary Material

Supplemental material is available online at www.g3journal.org/lookup/suppl/doi:10.1534/g3.116.038539/-/DC1.
