# Supplementary figures and images for: CRISPR-Cas-Induced Mutants Identify a Requirement for dSTIM in Larval Dopaminergic Cells of Drosophila melanogaster
Source: G3 (Bethesda). 2017 Jan 26;7(3):923–33. doi: 10.1534/g3.116.038539 (PMC5345722; doi:10.1534/g3.116.038539)

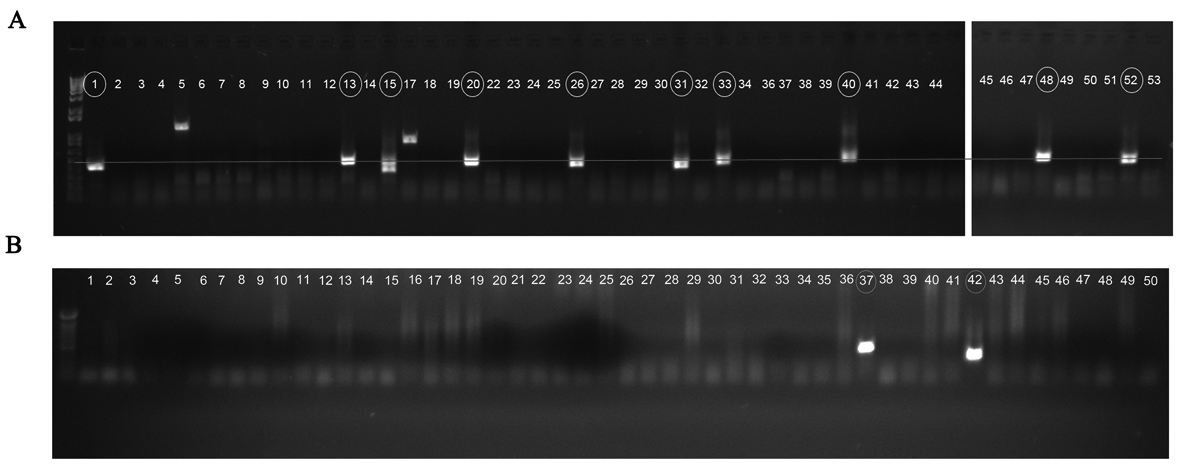

Supplement: Supplementary file 1 [file 923FigureS1.tif]

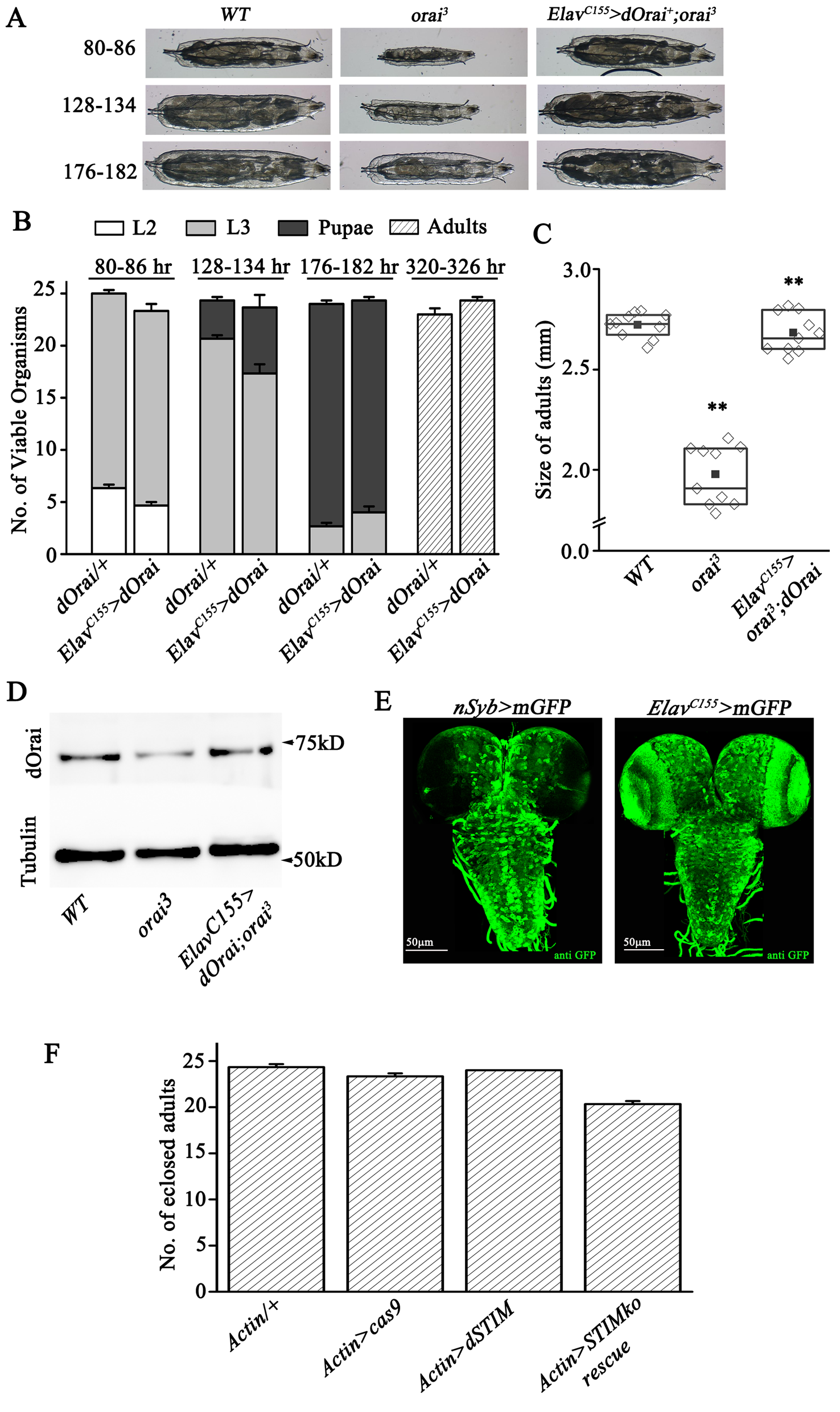

Supplement: Supplementary file 2 [file 923FigureS2.tif]

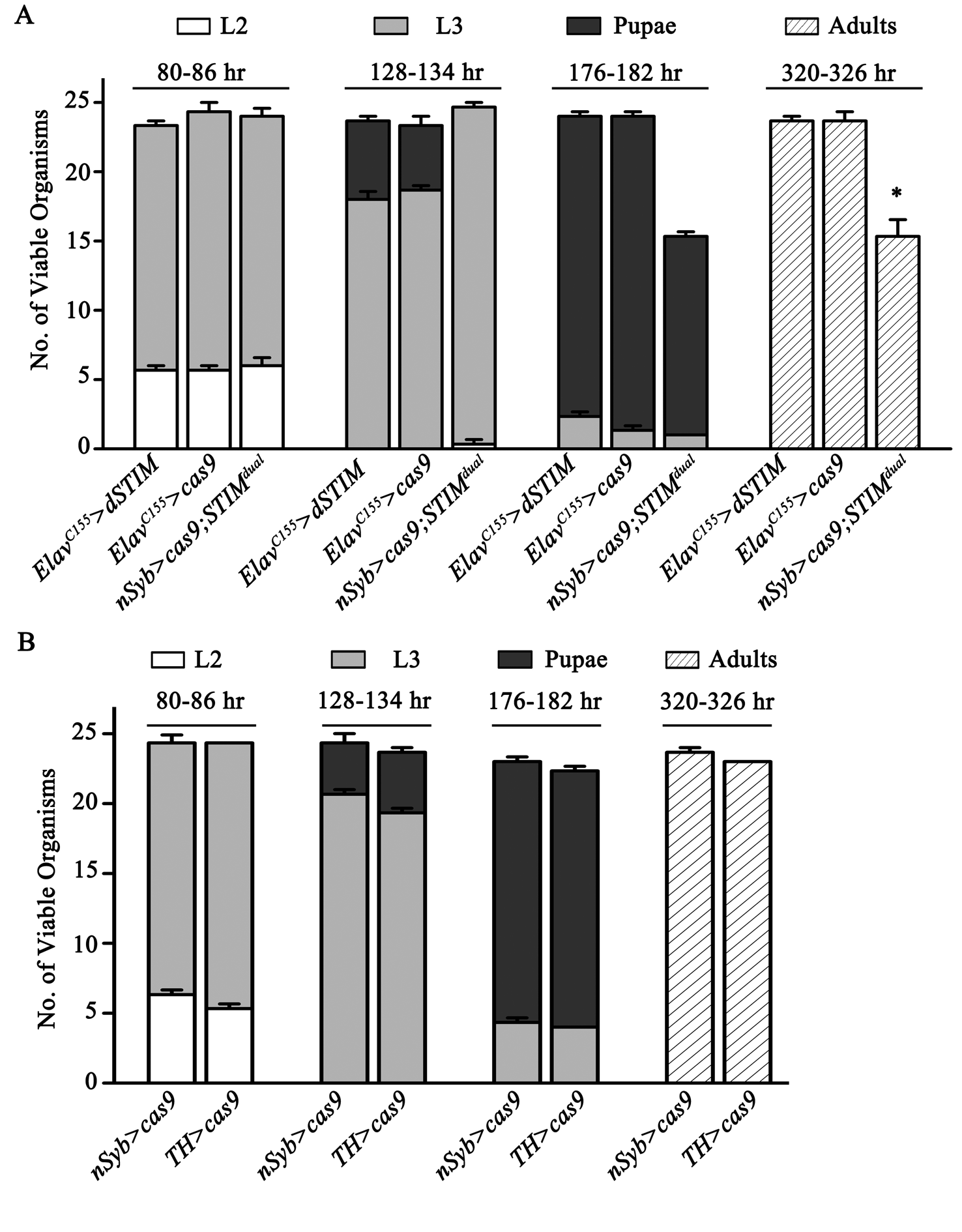

Supplement: Supplementary file 3 [file 923FigureS3.tif]

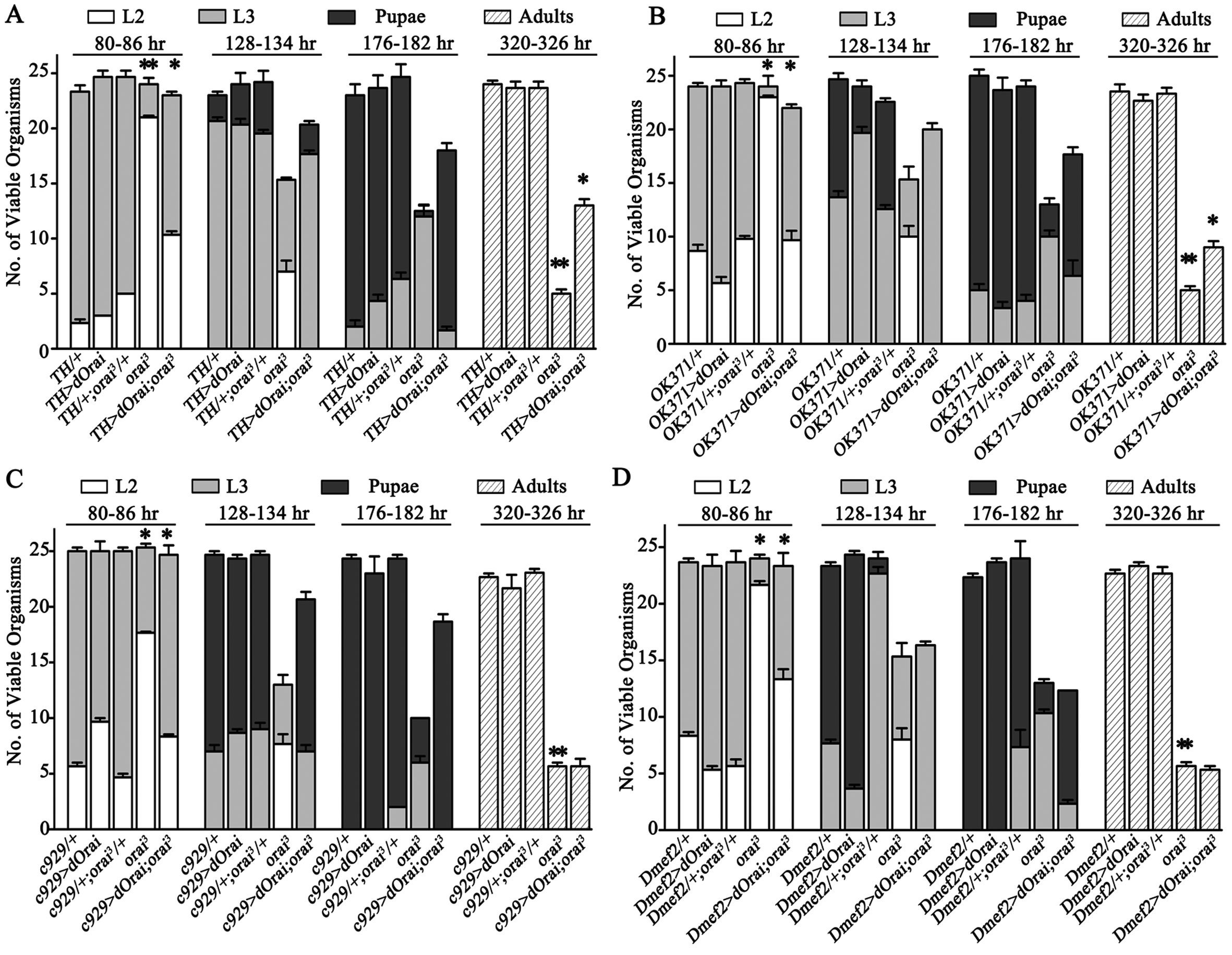

Supplement: Supplementary file 4 [file 923FigureS4.tif]

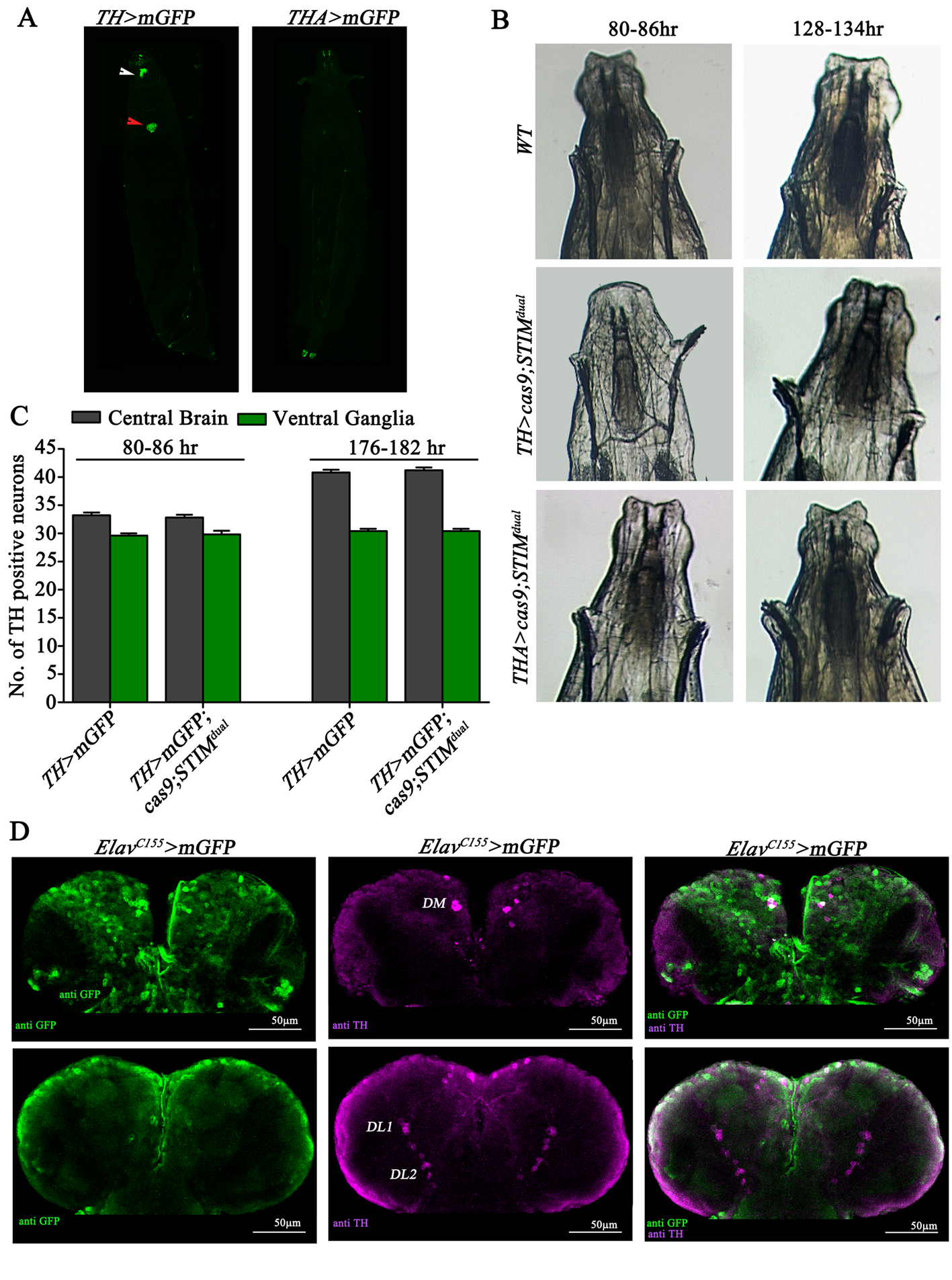

Supplement: Supplementary file 5 [file 923FigureS5.tif]
