# Supplementary material for: CRISPR-Cas-Induced Mutants Identify a Requirement for dSTIM in Larval Dopaminergic Cells of Drosophila melanogaster
Source: G3 (Bethesda). 2017 Jan 26;7(3):923–33. doi: 10.1534/g3.116.038539 (PMC5345722; doi:10.1534/g3.116.038539)
Supplement: Supplementary file 6 [file 923FileS1.pdf]

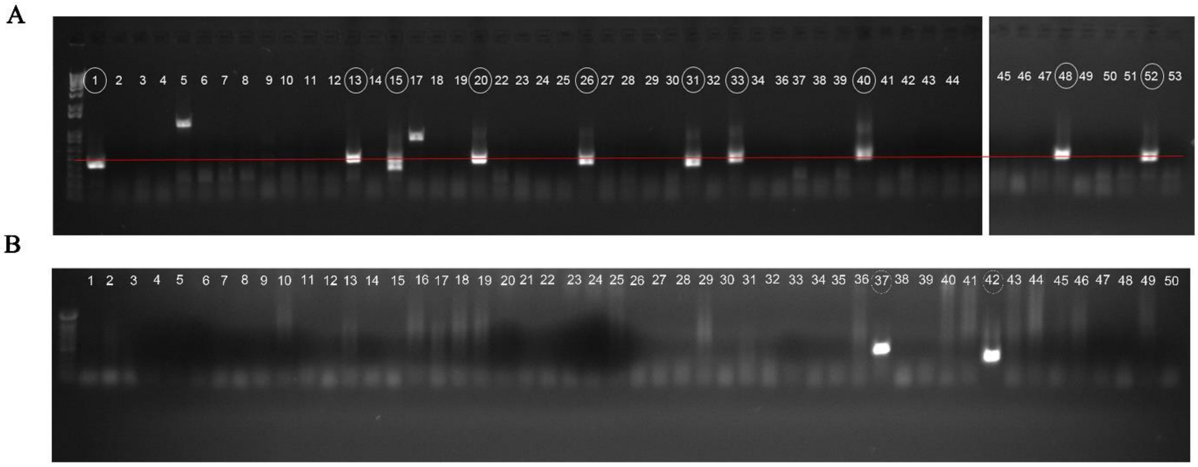

Figure S1- Two positive *STIMko* lines were obtained. (A) Gel showing PCR of 72 viable F0 adults, out of which 17 lines were positive and taken as founder lines (1,13,15, 20, 28, 31, 33, 40, 48 and 52) marked by circle. (B) PCR confirmation of putative *STIMko* lines from 198 F1 progenies from 17 founder lines. Two lines (37 and 42 marked by circle) were positive for *dSTIM* deletion.

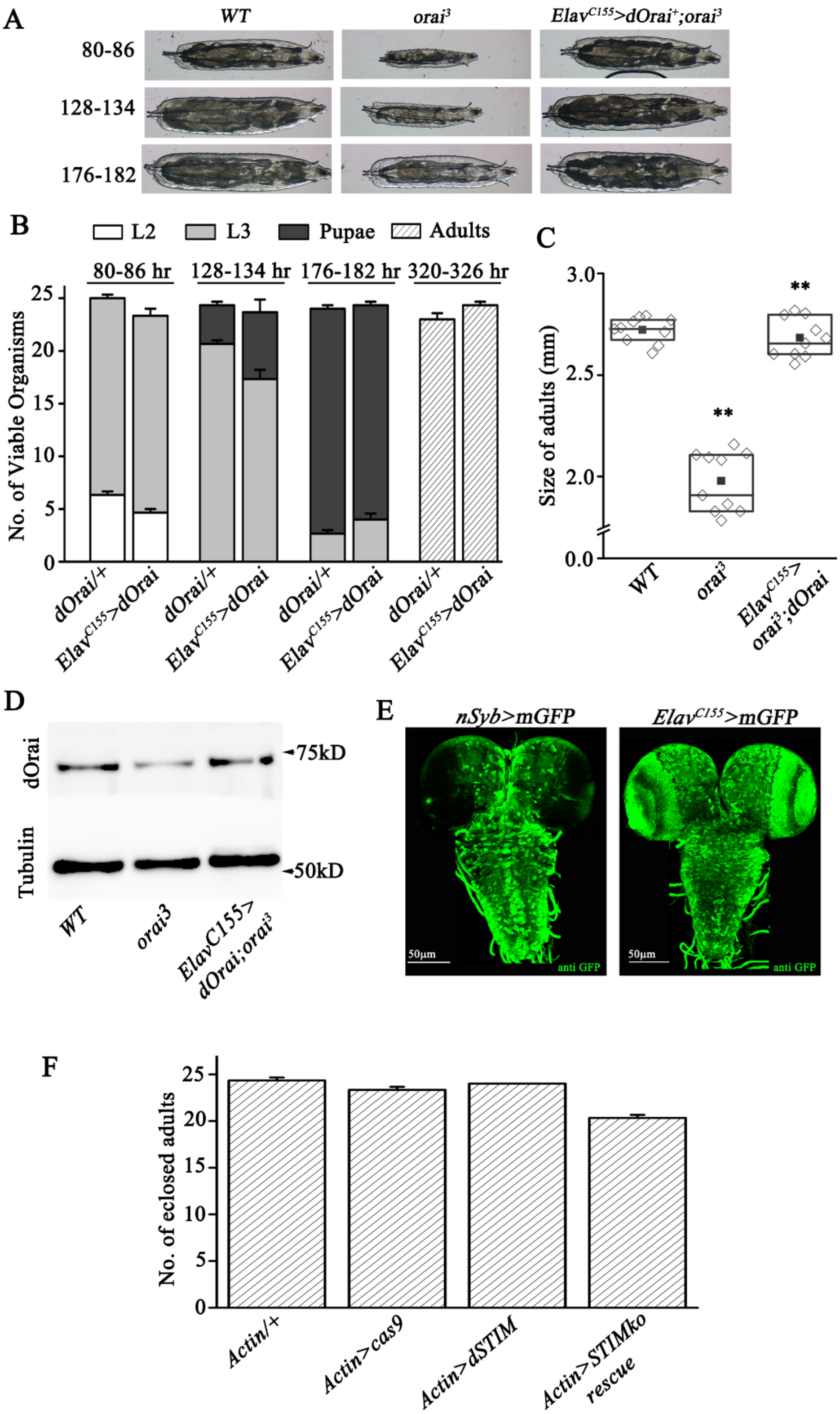
A
WT
orai3
ElavC155>dOrai+;orai3
80-86
128-134
176-182
B
L2
L3
Pupae
Adults
80-86 hr
128-134 hr
176-182 hr
320-326 hr
No. of Viable Organisms
dOrai/+
ElavC155>dOrai
C
Size of adults (mm)
**
WT
orai3
ElavC155>
orai3;dOrai
D
dOrai
Tubulin
75kD
50kD
WT
orai3
ElavC155>
dOrai;orai3
E
nSyb>mGFP
ElavC155>mGFP
50µm
anti GFP
F
No. of eclosed adults
Actin/+
Actin>cas9
Actin>dSTIM
Actin>STIMko
rescue

Figure S2- Pan-neuronal overexpression of *dOrai* did not result lethality. (A) $orai^3$ homozygous larvae were smaller in size compared to the control. Pan-neuronal overexpression of *dOrai* rescued the larval size of $orai^3$ homozygotes compared to the control. (B) $Elav^{C155}$>*dOrai* organisms did not show larval lethality and developmental defect. (C) The box plot represents the size of adult *Drosophila* of indicated genotypes. Each diamond shaped point represents size of an individual organism. $orai^3$ flies were significantly smaller than controls (n=10, Student's t-test **P<0.001). The error bars represent standard error of means. (D) A western blot for dOrai in lysates of CNS obtained from third instar larvae of the indicated genotypes. Pan-neuronal overexpression of *dOrai* ($Elav^{C155}$>*dOrai*;$orai^3$) rescues the reduced expression of Orai in $orai^3$ larval brains. (E) Expression patterns of *nSybGAL4* and $Elav^{C155}$ *GAL4* strains in 176-182 hr larval CNS, marked by GFP. (F) Ubiquitous expression of *UASdSTIM* in the *STIMko* background with *Actin5cGAL4* rescued viability to a large extent (compare with no adults seen in Figure 2B). Larvae were collected at 60-64hr AEL and number of adults that eclosed were quantified from three independent batches of 25 larvae each.

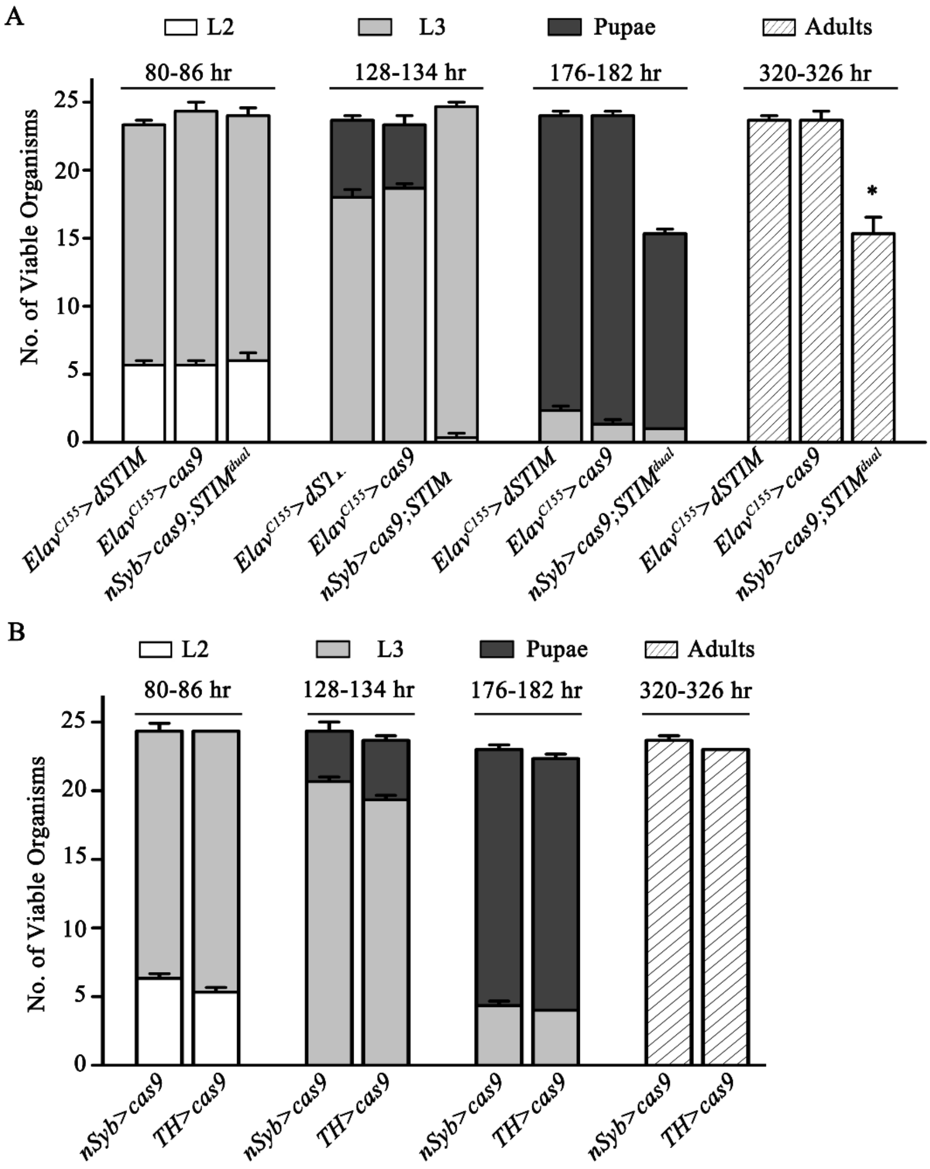

Figure S3- Pan-neuronal knockout of *dSTIM* with *nSyb-GAl4* resulted in partial lethality. (A) Pan-neuronal overexpression of *dSTIM* (*Elav^{C155}>dSTIM*) and *cas9* (*Elav^{C155}>cas9*) did not result in larval lethality or developmental delay. Pan-neuronal knockout of *dSTIM* with *nSybGAL4* resulted in partial lethality of *nSyb>cas9;STIM^{dual}* organisms. (B) Animals with *nSybGAL4* and *THGAL4* driven expression of *cas9* were viable with no developmental delays.

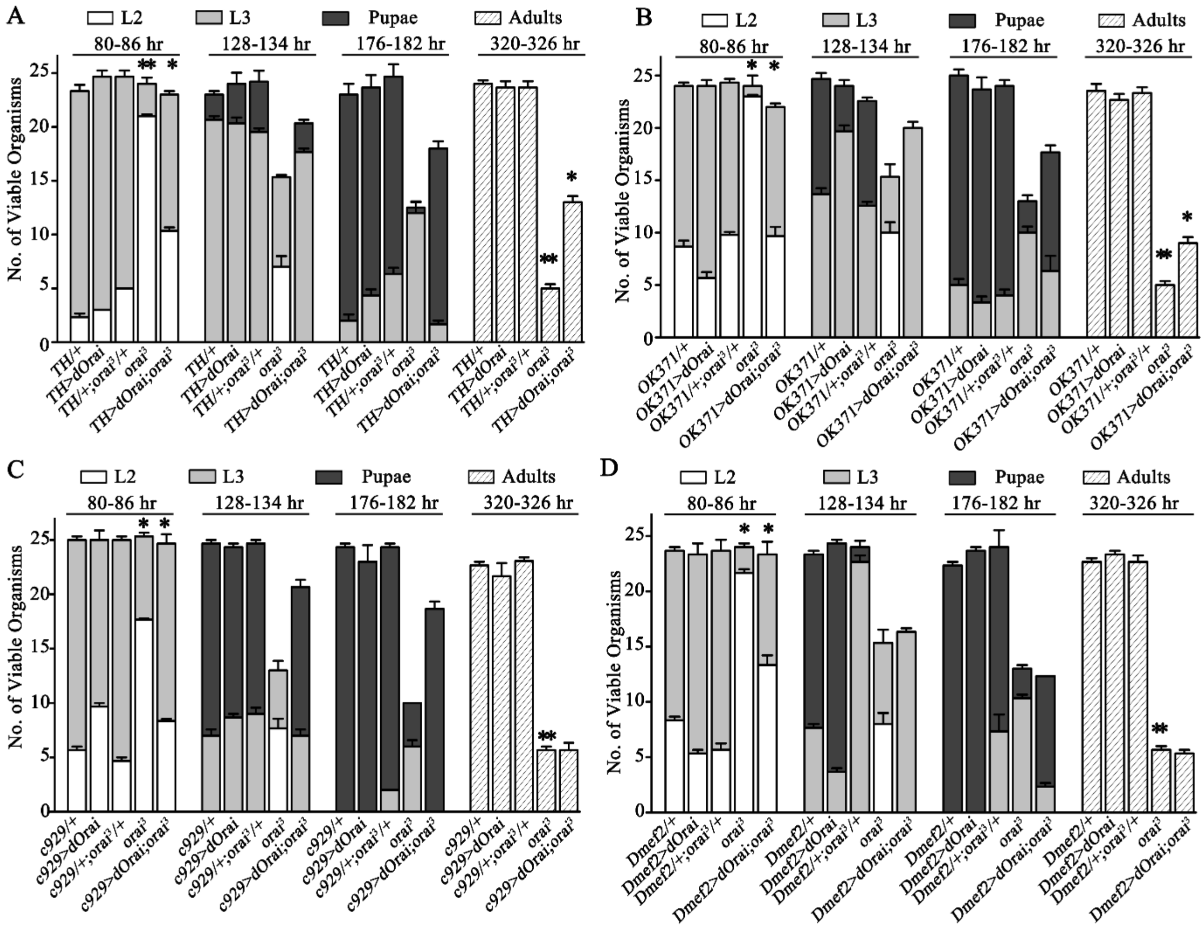

Figure S4- Overexpression of *dOrai* in neurons partially rescued the lethality of *orai³* homozygous organisms. Overexpression of *dOrai* in dopaminergic neurons (*TH>dOrai;orai³*) partially rescued lethality of *orai³* homozygotes. (B) Overexpression of *dOrai* in glutamatergic neurons (*OK371>dOrai;orai³*) partially rescued lethality of *orai³* homozygotes. (C, D) Overexpression of *dOrai* either with *c929* or *Dmef2 GAL4* did not rescue lethality of *orai³* homozygotes. Comparisons of number of second instar larvae at 80-86hr and adults at 320-326hr are in Table 3.

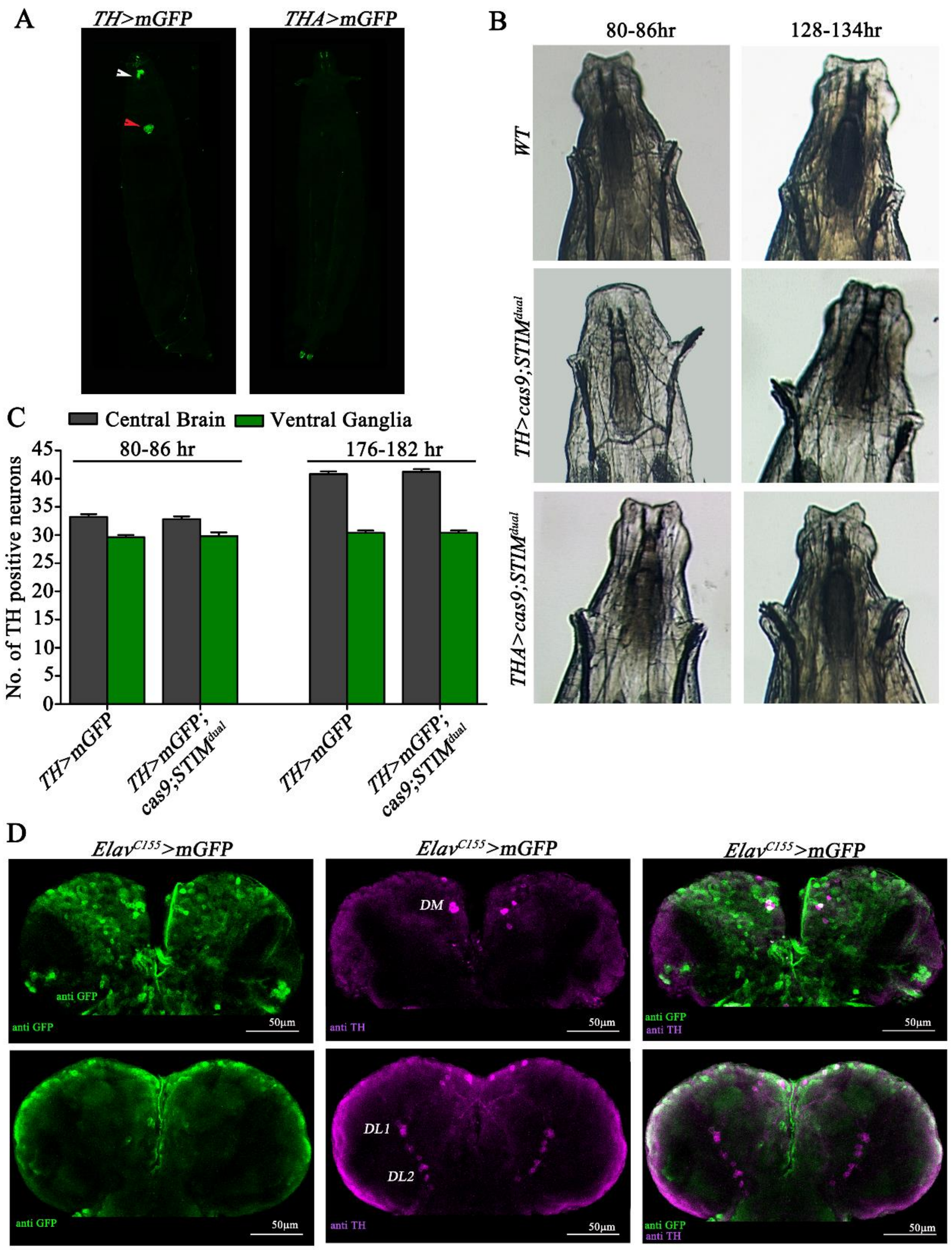

A
TH>mGFP
THA>mGFP
B
80-86hr
128-134hr
WT
TH>cas9;STIMdual
THA>cas9;STIMdual
C
Central Brain
Ventral Ganglia
80-86 hr
176-182 hr
No. of TH positive neurons
TH>mGFP
TH>mGFP; cas9;STIMdual
D
ElavC155>mGFP
DM
DL1
DL2
anti GFP
anti TH
50μm

Figure S5- Effect of dSTIM knockout in dopaminergic cells (A) Expression pattern of *TH* (*TH>mGFP*) and *THAGAL4s* (*THA>mGFP*) in third instar larvae. White arrowhead indicates the CNS and the red arrowhead indicates the Proventriculus. Both *TH* and *THAGAL4* mark the hypoderm. *THAGAL4* does not mark the CNS and the proventriculus. (B) Images of mouth hooks from third instar larvae at 80-86hr and 128-136hr AEL. *STIM* knockout either with *TH* (*TH>cas9;STIM^{dual}*) or *THA* (*THA>cas9;STIM^{dual}*) *GAL4* did not affect development of mouth hooks. (C) Number of TH GAL4 marked cells in the central nervous system at the indicated stages of development. Knock out of *dSTIM* from dopaminergic cells did not result in loss of neurons. Average number of TH positive neurons were the same in the central brain and ventral ganglia of both *TH>mGFP* and *TH>cas9;STIMdual* organisms either at 80-86hr or 176-182hr. N= 5 brains of each genotype. (D) Immunostaining of the central brain from early third instar larvae of *Elav^{C155}>mGFP* organisms with anti-GFP and anti-TH antibodies. *Elav^{C155}>mGFP* expression overlaps with anti-TH immunostaining in Dorso-medial neurons (DM) present in stacks numbers 20-30. However, Elav driven GFP expression does not overlap with anti-TH immunostaining of Dorso-lateral neurons (DL1 and DL2) present in stack numbers 46-55.
